# Rapid and Efficient Isolation of Exosomes by Clustering and Scattering

**DOI:** 10.3390/jcm9030650

**Published:** 2020-02-28

**Authors:** Jinhyun Kim, Hoyoon Lee, KyongHwa Park, Sehyun Shin

**Affiliations:** 1 School of Mechanical engineering, Korea University, Seoul 02841, Korea; mimoz742@korea.ac.kr (J.K.); leehoyoon@korea.ac.kr (H.L.); 2 Department of Internal Medicine, Anam Hospital, Korea University, Seoul 02841, Korea; khpark@korea.ac.kr; 3 Engineering Research Center for Biofluid Biopsy, Seoul 02841, Korea

**Keywords:** tumor-derive extracellular vesicles, isolation, liquid biopsy, blood, polymer

## Abstract

Tumor-derived extracellular vesicles (EVs) have become important biomarkers of liquid biopsies for precision medicine. However, the clinical application of EVs has been limited due to the lack of EV isolation practical technology applicable to clinical environments. Here, we report an innovative EV isolation method, which is quick and simple, and facilitates high-yield and high-purity EV isolation from blood. Introducing a cationic polymer in plasma resulted in rapid clustering of anionic EVs and a chaotropic agent can separate EVs from these clusters. Isolated EVs were characterized in terms of size distribution, morphology, surface protein markers, and exosomal RNA. Through performance comparison with various methods, including ultracentrifugation (UC), the present method delivered the highest recovery rate (~20 folds that of UC) and purity ratio (3.5 folds that of UC) of EVs in a short period of time (<20 min). The proposed method is expected to be used in basic and applied research on EV isolation and in clinical applications.

## 1. Introduction

Exosomes are nanosized vesicles (30–220 nm) extracellular vesicles (EVs), which, play a significant role in the delivery of signaling molecules for cell–cell communication [1,2,3,4] and in the mediation of pathological signaling [5,6,7]. EVs including exosomes are shed from cells into body fluids such as blood and urine. A main feature of EVs is the internal molecules such as messenger RNA (mRNA), micro RNA (miRNA), non-coding RNA, and genomic DNA), proteins, metabolites, and lipids [1,2,3,4]. Owing to the protein-lipid membrane of EVs, internal nucleic acids are securely preserved in body fluids [1,2]. Tumor-derived exosomes have been extensively studied for potential applications of early detection of cancer and therapeutic monitoring [8,9]. Despite the potential significance of exosomes, they remain to be challenging analytes mainly due to the lack of EV isolation technology. The technical difficulty in EVs isolation arise from their unique characteristics such as nanoscale size, near neutral buoyancy, and containing excessive proteins and lipids in body fluids [10].

Ultra-centrifugation (UC) is the most widely used method in research environments, even though it is highly labor-intensive, time-consuming, and poor performance of yield [11,12,13,14]. A size-based isolation method has been developed for EV isolation. Ultrafiltration (UF) using multistep filtration is one of the popular technique to isolate EVs based on their targeted molecular weight or size using membrane filters [15,16,17,18]. The filtration method still suffers from the clogging problem in the filter, which deteriorates the performance of isolation. However, recent studies showed significant advances in purity, yield, and easy operation of isolating EVs [17,18]. Other methods for isolating EVs, such as affinity-based methods, require specific antibodies for surface proteins of EVs [19,20]. A typical technique is the immuno-magnetic beads to capture exosomes, which provides a highly specific isolation of EVs by tapping on immune-affinity interactions between those proteins (antigens) and their antibodies. Unfortunately, surface biomarkers for various cancer have not been fully elucidated and also would be multiple.

EV precipitation using polymers is a commonly used method of commercial products such as ExoQuick^TM^ (SBI). The water-excluding polymers such as polyethylene glycol (PEG) can tie up water molecules and force less soluble components out of solution [21]. Then, EVs can be isolated via centrifugation at low *g*-forces. This method can significantly reduce the total cost of the experiment and simplify complicated workflows by replacing UC with a common centrifuge equipment. However, the polymer-based precipitation method suffers from low purity and moderate yield [14], the low-purity problem is the result of co-precipitation of proteins in a sample because PEG decreases the solubilities of both EVs and proteins [15]. Such protein-contaminated EV isolation is detrimental to downstream analyses such as exosome protein analysis. Thus, the purity of exosomes delivered by the polymer-based precipitation method must be further improved. Meanwhile, the rapid development of microfabrication technology has progressed to provide various techniques of exosome isolation such as acoustic [22,23] and electrophoretic [24,25] manipulations. Even though these techniques are at the research level, they are expected to significantly reduce sample volume, reagent consumption and separation time.

The brief review of the currently available methods and techniques of EV isolation reveals that there are still unmet needs for exosome isolation. Here, we introduce a novel exosome isolation method, the exosome clustering and scattering, which provides high yield and purity, as well as rapid and easy operation. The method is a hybrid scheme of size-, charge-, and chaotic-based mechanisms. First, we demonstrate that the present method can efficiently isolate exosomes from plasma. Then, we examined the characteristics of the isolated EVs using nanoparticle tracking analysis (NTA), scanning electron microscopy (SEM) images, cryo-transmission electron microscopy (cryo-TEM) images, Western blot assay, BCA protein assay, and RNA assay. Further, we compare the microRNA and proteomic profiles from EVs isolated by ExoCAP^TM^, ExoQuick^TM^, exoEasy^TM^ and standard UC, demonstrating that results from our method is superior to other methods in terms of yield, purity, operation time, and easiness.

## 2. Materials and Methods

### 2.1. Working Principle of the Clustering-and-Scattering Method

**Figure 1:**
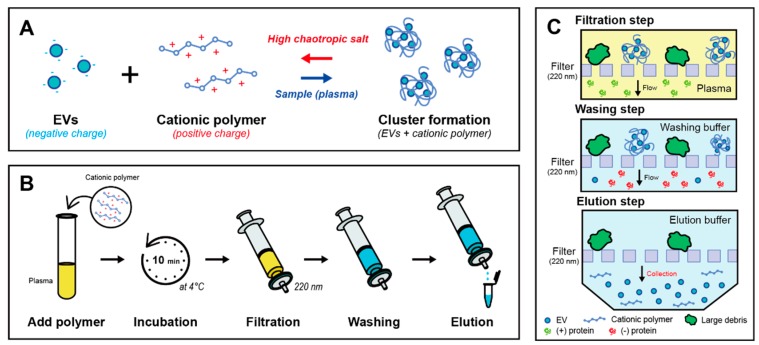
Schematic illustration of PLL-clustering method for extracellular vesicle isolation. (**A**) Cluster formation by adding cationic polymer (PLL) in plasma sample and scattering EVs in elution buffer with chaotropic salt. (**B**) Experimental procedure to isolate EVs from plasma sample using a syringe and a filter. (**C**) Simple filtration, washing, and elution steps for EV isolation.

The present method uses a hybrid scheme of size-, charge-, and chaotic-based mechanisms to isolate EVs from body fluids (Figure 1A). By adding the cationic polymer to the plasma, negatively charged EVs aggregated with the polymer because of charge interaction. The mixture of plasma and PLL solution was incubated at 4 °C for 10 min (Figure 1B). Clustered EVs with the polymer formed several hundred nanoscale particulates. Then, the mixture was filtered using a syringe filter with a pore size of 220 nm; large clustered particulates remained on the filter and other small fragments and proteins were flushed out (Figure 1C). To recover EVs from the PLL clusters, a chaotropic agent such as guanidium thiocyanate (GuTc) was introduced to the EV clusters. Due to the characteristic of denaturizing proteins, captured EVs were released from the PLL clusters. Two different concentrations (2 M and 5 M) of GuTc solutions were used as washing buffer (2 mL) and elution buffer (200 μL), respectively. The PLL polymers were captured on an anionic membrane and then purified and concentrated EVs were successfully isolated.

The entire process including incubation, washing, and elution was completed within 20 min with high yield and purity. The isolated EVs are then used for downstream physical characterization and molecular analysis. The particle diameter was measured using dynamic light scattering (DLS). 

### 2.2. Blood Sample Preparation

This study was conducted according to the principles of the Declaration of Helsinki. The Institutional Review Board of Korea University Anam Hospital, Seoul, Republic of Korea, approved the study protocol (IRB project number: 2016AN0090). All participants provided written consent for the samples. Blood samples were collected in 3-mL K2-EDTA vacutainers (Becton Dickinson, Franklin Lakes, NJ, USA) from the median cubital vein. Plasma was isolated after centrifuging whole blood at 1900× *g* for 10 min using a model 1248 apparatus (LABOGEN, Denmark), followed by centrifuging at 12,000× *g* for 15 min. Then, the plasma was filtered with an 800-nm pore-size mesh to remove large debris. The volume of each plasma sample was 1 mL.

### 2.3. Isolation of EVs

#### 2.3.1. Ultracentrifugation

UC is a traditional yet widely used gold standard method of isolating EVs. The isolation protocol is simple yet time consuming (t > 6 h) and labor intensive. In this study, plasma and phosphate buffered saline (PBS) were mixed with a 1:1 ratio, and the mixture was centrifuged to remove residual cellular components (4 °C, 12,000× *g*, 30 min). The supernatant was transferred, and centrifugation was repeated once under the same conditions. The supernatant was filtered using a 220-μm pore-size syringe filter (Merck Millipore, Burlington, MA, USA), followed by ultracentrifugation (CP100WX; Hitachi, Tokyo, Japan) at 120,000× *g* and 4 °C for 2 h. After aspirating the supernatant, the pellet at the bottom was carefully washed with PBS at 120,000× *g* and 4 °C for 1 h and then finally resuspended in 50 μL of PBS.

#### 2.3.2. EV Precipitation with Polymer

ExoQuick^TM^ exosome precipitation solution (EXOQ5A-1; System Biosciences, Palo Alto, CA, USA) was selected as a representative commercial product for isolating EVs. The standard protocol is described in the ExoQuick User Manual. Briefly, the plasma sample was mixed with the ExoQuick solution, which is a PEG-based solution. The mixture was incubated for 30 min at 4 °C. After incubation, the mixture was centrifuged at 1500× *g* for 30 min, and the supernatant was carefully aspirated while leaving the pellet at the bottom. After additional centrifugation at 1500× *g* for 5 min, all traces of the ExoQuick solution were removed and the remaining pellet was resuspended in 200 μL of PBS.

### 2.4. Analysis of Isolated Particles

#### 2.4.1. Zeta Potentials in EVs and Polycationic Polymer

The zeta potentials of EVs isolated using UC and the PLL solution (10 mg/mL), as well as those of clusters with PLL, were measured using a zeta potential analyzer (Zetasizer Pro; Malvern Panalytical, Malvern, UK). Since the cluster was difficult to resuspend in deionized water, a clustered pellet from 1 mL of plasma (PLL 0.5 mg/mL) was first resuspended in 10 μL of GuTc solution (10 M); the solution was then added to 990 μL of deionized water. The mixture was carefully dispersed for 5 min using a vortex mixer.

#### 2.4.2. Cryo-Transmission Electron Microscopy (Cryo-TEM) Images

The EVs isolated using UC and the PLL method were transferred to a 20 nm-mesh grid. The grids were then subjected to freezing incubation (−196 °C, 2 h) using Vitrobot^TM^ (FEI, Hillsboro, OR, USA). After all samples were prepared, EVs were observed with TEM (Tecnai G2-F20, FEI, Hillsboro, OR, USA).

#### 2.4.3. Scanning Electron Microscopy (SEM) Images

After UC of the plasma sample, the sample was filtered using an anodic aluminum oxide (AAO) membrane mounted in a gasket. Also, the PLL clustered sample was filtered using another AAO membrane. These membranes were incubated in glutaraldehyde solution (Sigma-Aldrich, St. Louis, MO, USA) for 30 min. The membranes were sequentially rinsed with 25%, 50%, 75%, 90%, and 100% ethanol and incubated overnight at 37 °C in a dry oven. After coating the membrane with Pt, EVs and clusters subjects existing on the membranes were observed by SEM (Quanta 250 FEG; FEI, Hillsboro, OR, USA).

#### 2.4.4. Nanoparticle Tracking Analysis (NTA)

For each NTA analysis, 1 mL of EV solution isolated using UC, ExoQuick, exoEasy, or the PLL clustering method was used. Briefly, a PBS-diluted sample was placed in the assembled sample chamber of the NanoSight LM10 system (Malvern Panalytical, Westborough, MA, USA), and the microparticles were focused using the fingerprint area as a reference. Video images of the EVs were acquired, and their mean size and concentration were analyzed according to each dilution factor. The experiments were independently replicated three times.

### 2.5. Analysis of Proteins and Nucleic Acids

#### 2.5.1. Western Blot Analysis

Protein samples were separated by gel electrophoresis using SDS-PAGE Mini-PROTEAN^®^ TGX™ Precast Gel (456-1035; Bio-rad, Hercules, CA, USA) and subjected to immunoblotting with rabbit polyclonal antibodies (1:2000 dilutions) anti-CD9 (ab92726), anti-CD81 (ab109201), anti-ALIX (ab186429), anti-TSG101 (ab125011), and Goat Anti-Rabbit IgG H and L (HRP) (ab205718) (Abcam, Cambridge, UK). The protein bands were analyzed using an enhanced chemiluminescence (ECL) reagent and ChemiDoc™ XRS + System (Bio-Rad, Hercules, CA, USA).

#### 2.5.2. Protein Contamination Assay

A Pierce^TM^ BCA protein assay kit (#23225; Thermo Scientific, Waltham, MA, USA) was used for a purity test. A standard curve (range 0–2000 μg/mL) was derived with nine points of serial dilution with bovine serum albumin (BSA) and a working reagent. All samples and standard points were replicated three times. The samples (100 μL each) were mixed with 2.0 mL of working reagent and incubated at 37 °C for 30 min. After cooling to room temperature, each absorbance difference, which was subtracted by averaged absorbance of blank standard replicates at 562 nm, was measured by a spectrometer (DS-11; Denovix, Wilmington, DE, USA), and the absorbance differences were converted to μg/mL via the standard curve. If a protein concentration exceeded the upper limit of the standard curve of 2000 μg/mL, the sample was diluted until it could be measured within the standard range, and the final concentrate was calibrated considering the dilution factor.

#### 2.5.3. RNA Analysis

RNA was isolated using the seraMir Exosome RNA purification kit (RA806A-1, SBI, Palo Alto, CA, USA). All isolation processes were performed according to the manufacture’s protocol. To quantify EV miRNA markers, the RNA eluate solution was subjected to reverse transcription with the TaqMan MicroRNA RT kit (4366596, Life Technologies, Eugene, OR, USA) and TaqMan Micro RNA Assays (4427975, Life Technologies, USA). TaqMan Universal Master Mix II, no UNG (4440040, Life Technologies, Eugene, OR, USA) was used, together with the miRNA assays hsa-let-7a-5p, ID 000377, and hsa-miR-142-3p, ID 000464. Further experiments were performed with RNA eluate using the Agilent Eukaryote Total RNA Pico chip on an Agilent 2100 bioanalyzer (Agilent Technologies, Santa Clara, CA, USA).

## 3. Results

### 3.1. Cationic Polymer-Induced EV Cluster Formation

**Figure 2:**
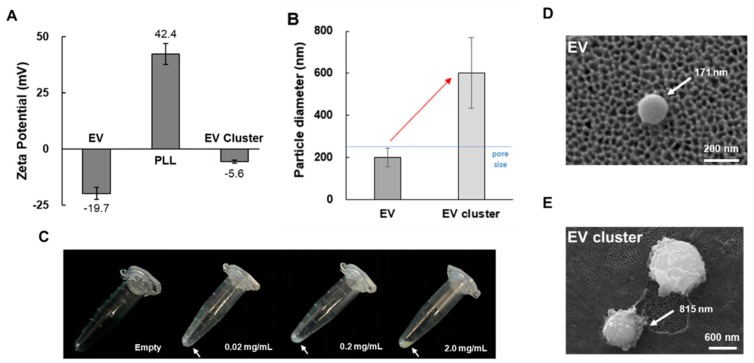
Cluster formation of EVs in plasma sample. (**A**) Zeta potential of EVs isolated by UC and PLL solution (10 mg/mL), and EV cluster obtained from UC sample. (**B**) Particle diameter of pure EV obtained by UC and EV cluster. (**C**) Images of precipitated pellet formation with varying PLL concentrations (0–2.0 mg/mL) with plasma sample. Incubation time was fixed at 1 h. (**D**) SEM images of EV isolated by UC and (**E**) EV clusters.

Figure 2 presents the zeta potentials of the EVs, the PLL polymer solution, and the EV clusters isolated via centrifuging. The EVs showed a negative charge of −19.7 mV, whereas the PLL solution (10 mg/mL) had a strong positive zeta potential charge of 42.4 mV. When the EVs were combined with the PLL polymer, the zeta potential of the EV–PLL clusters was neutralized to −5.6 mV. These results imply that there was a strong electrostatic interaction between the negative charge of the EVs and the positive charge of the PLL polymers. We compared the sizes of particles before and after adding the PLL solution to the plasma. The particle diameter was measured using dynamic light scattering (DLS). As shown in Figure 2B, the size of the EV cluster (600 nm) was distinctly larger than that of the EVs (200 nm). This also implies that EVs were clustered using PLL.

To check the formation of EV–PLL clusters, the plasma–PLL polymer mixture was incubated at 4 °C for 10 min and then centrifuged. The resulting pellets in a tube are shown in Figure 2C. At room temperature, EV clustering was not as efficient as it was at 4 °C (Figure S1). As the concentration of PLL increased, the pellet size and shape also increased, and the pellets became more vivid. Isolated EVs and clustered EVs with PLL polymer were also visualized with SEM as shown in Figure 2D,E.

In the clustered EVs, the EVs were completely encapsulated with polymer, and the number of EVs per cluster could not be determined. However, the SEM images provided the sizes of the EVs and EV clusters, which were 171 nm and 815 nm, respectively.

### 3.2. Resuspension of EV Clusters Using Chaotropic Salt

**Figure 3:**
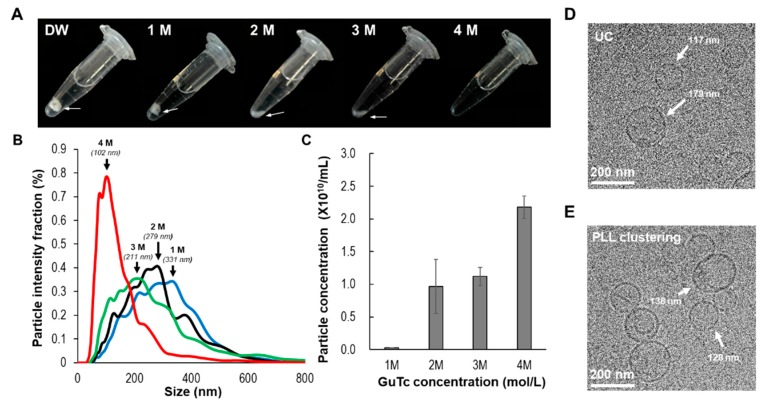
Resuspension of EV cluster in GuTc buffer. (**A**) Serial images of resuspension of EV cluster according to GuTc concentration. PLL was fixed at 0.2 mg/mL and GuTc concentration was varied from 0 to 4 M. (**B**) Effect of GuTc concentration during elution according to particle size (black arrows indicate peak size) and (**C**) concentration analyzed using NTA. Cryo-TEM images of EVs isolated by (**D**) UC and (**E**) PLL (0.2 mg/mL) and GuTc (4 M). (PLL: poly-l-lysine, GuTc: guanidium thiocyanate).

EV clusters were successfully acquired by the simple addition of PLL solution to plasma. To isolate EVs for subsequent applications, we needed to resuspend the EV clusters in a proper buffer. However, strongly aggregated polymers and EVs could not be suspended in deionized water (Figure 3A) and PBS solution. Therefore, we used a chaotropic agent to control the solubility of the precipitate. In this study, we selected guanidium thiocyanate (GuTc) as a chaotropic agent to dissolve the PLL and EVs. As shown in Figure 3A, various concentrations of GuTc solution (0–4 M) were examined in terms of their ability to dissolve EV–PLL clusters. For a fixed PLL concentration in plasma (0.2 mg/mL), a 4 M solution of GuTc delivered the best performance among the examined concentrations to dissolve the clusters. The optimized GuTc buffer dissolved the EV cluster pellets within 1 min.

The sizes and particle concentrations of isolated EVs were analyzed using NTA with respect to GuTc concentrations, as shown in Figure 3B,C. As the concentration of GuTc solution increased, the size of the EVs gradually decreased. At low concentrations of GuTc (1 M, 2 M), larger particles were observed (~300 nm). The EV concentration was fairly low in 1 M GuTc, 0.03 × 1010, whereas at high concentrations of GuTc (4 M), the particle size significantly decreased, with a peak value of 102 nm, which might be considered fully dissolved from the clusters. Furthermore, the morphology of isolated EVs from PLL clustering was compared with that from UC in the TEM images as shown in Figure 3D,E. In both the methods, clear shapes of vesicles, 100–200 nm in diameter, were observed.

### 3.3. Western Blot Assay in PLL Clustering

**Figure 4:**
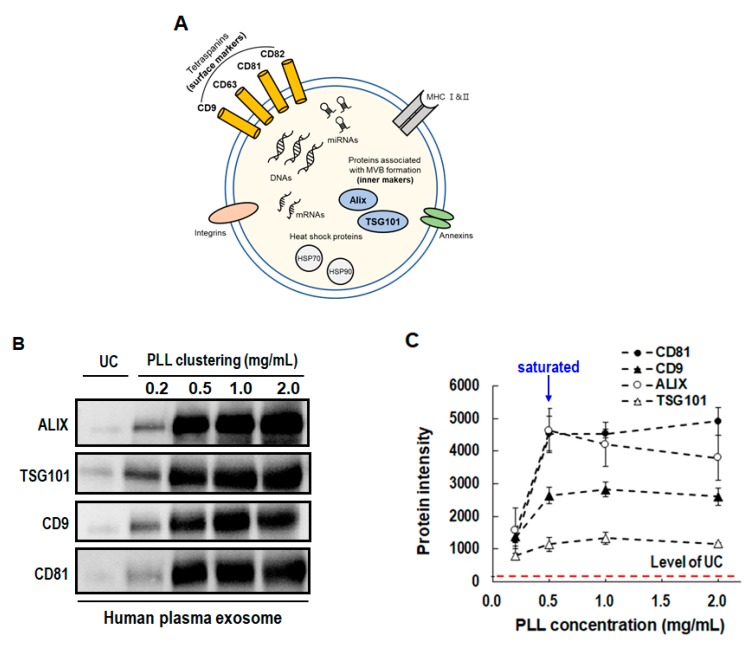
Detection of EV surface protein markers (CD81 and CD9) and inner protein markers (ALIX and TSG101) according to PLL concentration (0.2–2.0 mg/mL). (**A**) Diagram of molecular composition in EVs. (**B**) Band images of Western blot assay. (**C**) Band intensity of four EV markers. (PLL: poly-l-lysine, GuTc: guanidium thiocyanate).

We examined the protein markers of isolated EVs with varying PLL concentrations to find the optimal EV recovery. It is well known that EVs contain protein markers such as Alix (ALG-2-interacting protein X), TSG101 (tumor susceptibility gene 101 protein), HSP70 (heat shock protein), and the tetraspanins CD63, CD81, and CD9 [26,27,28]. Western blot assay was conducted for the protein analysis, and the results are shown in Figure 4. In the present experiment, four reference protein markers (ALIX, TSG101: inner protein markers; CD9, and CD81: Surface protein markers) were selected, as shown in Figure 4A, to characterize the isolated EVs. With the plasma input volume fixed at 1 mL and PLL concentration varying from 0.2 to 2.0 mg/mL, each protein level was compared. As shown in Figure 4B, there were clear bands of the four reference protein markers. This result indicates that the PLL clustering method successfully isolated EVs with the presence of surface and inner protein markers. Exosomal protein levels significantly increased when the PLL method was used, even at a relatively low concentration of 0.5 mg/mL; however, the levels did not increase with further increments in PLL concentration (1.0, 1.5, and 2.0 mg/mL), as shown in Figure 4C. It is worth noting that all the protein levels obtained by the PLL clustering method were higher than those obtained by UC for all PLL concentrations.

### 3.4. Purity and Yield in PLL Clustering

We analyzed the purity and yield of isolated EVs from our PLL method. Since human plasma includes various proteins having negative charge, the present PLL method based on a cationic polymer may have had contamination problems. In the present mechanism of the PLL clustering method, the strong cationic polymer, PLL, did not selectively bind with EVs but rather nonspecifically interacted with negatively-charged materials, such as cellular membranes, including EVs, cell debris, and even negatively-charged proteins. Thus, the PLL clustered particles shown in Figure 2E may include various proteins and cell debris, as well as EVs. However, larger cells and debris were not found in Figure 2E. Thus, the clusters included proteins, EVs, and PLL polymer.

**Figure 5:**
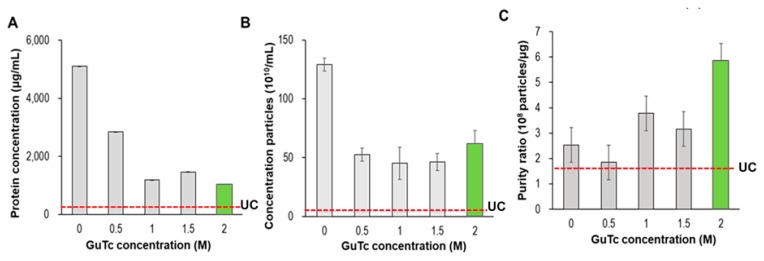
Comparison of GuTc concentration (0–2 M) in terms of (**A**) protein concentration, (**B**) particle concentration, and (**C**) purity ratio (particle/protein ratio).

The basic method of eliminating proteins from EVs and improving the purity of the isolated EVs involves adding a washing step. To identify the amount of plasma proteins that were removed after washing, a bicinchoninic acid (BCA) protein assay was performed. The amount of residual proteins in the final EV eluate extracted from 1 mL of plasma was measured according to the GuTc washing buffer (0–2 M), as shown in Figure 5. The final elution buffer was fixed as a 5 M GuTc solution (0.2 mL). After washing with GuTc solution, the amount of residual protein decreased drastically with an increase in GuTc concentration in the washing buffer. Since the PLL clusters barely dissolved in deionized water, the washing effect with deionized water alone was poor; the final eluent had 5100 μg/mL of residual proteins, as shown in Figure 5A.

Considering Figure 5A,B, the residual proteins were effectively removed by washing with a chaotropic agent, whereas the washing effect seemed to be saturated at 1.0 M GuTc concentration. Both the residual proteins and the concentrations of the particles did not further vary significantly with increase in GuTc concentration beyond 1.0 M. To determine the optimized washing protocol for EV isolation, the ratio of particle concentration to residual protein, called the purity ratio, was further analyzed. The purity ratio considering both protein contamination and the loss of EVs during washing was calculated by comparing the ratio of vesicle counts to protein concentration [19]. As shown in Figure 5C, the 2 M GuTc solution delivered the highest purity ratio. Compared to UC, the PLL method showed more extensive contamination of proteins but much better EV recovery. As shown in Figure 5C, the best purity ratio of the PLL method (5.9) was approximately three times that of UC (1.7).

It is worth noting that for 0 M GuTc (washing with PBS only and eluting with a 5.0 M GuTc solution), the residual proteins reached the maximum value (5100 μg/mL), and the concentration of particles were also at a maximum value (129 × 10^10^/mL). In other words, there was nearly zero loss of proteins in the PBS washing step, and most of the proteins and EVs were dissolved in the eluent buffer. Thus, we may assume that the total amount of proteins captured in the PLL was 5100 μg/mL, which decreased to 1041 μg/mL after washing with a 2.0 M GuTc solution. In fact, we demonstrated that the EV-depleted plasma proteins were clustered with PLL (Figure S2). Then, we may infer, from Figure 5B, that the maximum particle concentration of 129 × 10^10^/mL decreased to 61 × 10^10^/mL (47% recovery from the clustered EVs).

### 3.5. Performance Comparison with Other Methods

The yield and purity of the EVs isolated using the proposed method were compared with those of the EVs isolated using the standard method (i.e., UC) and commercially available kits (ExoQuick^TM^ and exoEasy^TM^). ExoQuick is a widely used method that adopts polymer (PEG)-based precipitation and isolation of EVs, whereas exoEasy is a spin column type of EV isolation kit utilizing the electrical charge interaction between EVs and the membrane.

**Figure 6:**
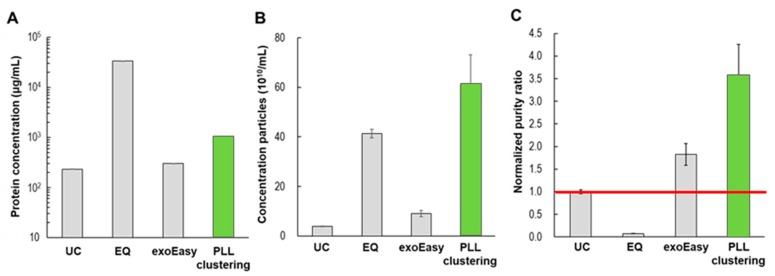
Comparison of EV isolation methods (UC, EQ, exoEasy, PLL) in terms of (**A**) protein concentration, (**B**) particle concentration, and (**C**) normalized purity ratio (particle/protein ratio). UC: ultracentrifugation, EQ: ExoQuickTM (SBI), exoEasyTM (Qiagen), PLL clustering: present study.

Similar to the previous BCA protein assay, we examined protein contamination with the different EV isolation methods, and the results are shown in Figure 6. The tested samples were the final eluents obtained in each method. The amounts of residual proteins in UC, exoEasy, and PLL clustering were 232 μg/mL, 301 μg/mL, and 1041 μg/mL, respectively, whereas that of ExoQuick was significantly higher (33,730 μg/mL). Considering the use of PEG, the high level of protein contamination in ExoQuick is not surprising [29]. However, in the analysis of EV recovery, both polymer-based methods (ExoQuick and PLL clustering) delivered high yields (41 × 10^10^/mL and 61 × 10^10^/mL, respectively), whereas UC and exoEasy delivered relatively low yields (3.8 × 10^10^/mL and 9 × 10^10^/mL, respectively). Figure 6C presents the normalized purity ratios for the four different methods, where UC was selected as the reference method. The proposed method, PLL clustering, and exoEasy delivered higher purity ratios than UC (3.5 and 1.8, respectively).

**Figure 7:**
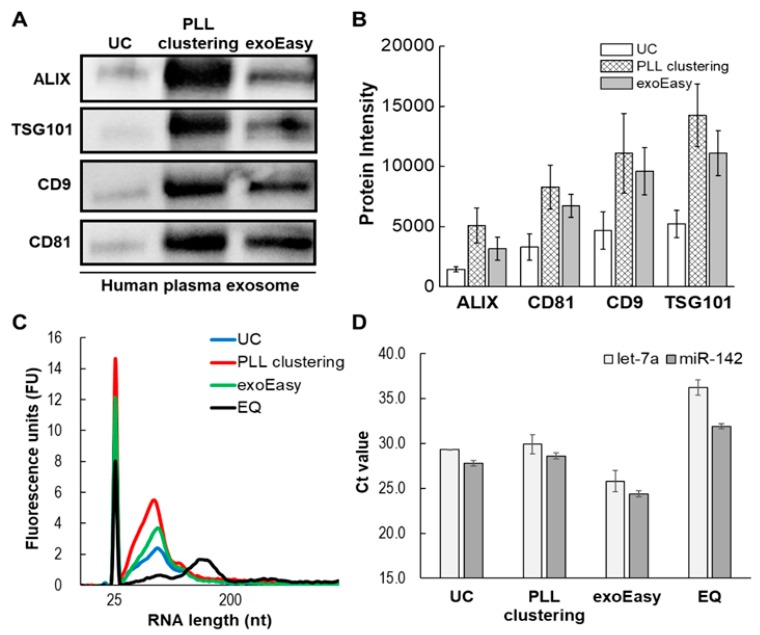
Comparison of EV isolation methods (UC, EQ, exoEasy, PLL clustering) via Western blot, bioanalyzer and RT-qPCR. (**A**) Band images of Western blot assay. (**B**) Protein level of four EV markers measured by band intensity. (**C**) Total EV RNA extracts analyzed on Agilent Eukaryote Total RNA Pico chips using a bioanalyzer. (**D**) EV miRNAs (hsa-let-7a-5p, hsa-miR-142-3p) were confirmed by RT-qPCR.

The EV protein markers from the three different methods (UC, PLL clustering, exoEasy) were examined with the Western blot assay, as shown in Figure 7A. The overall level of protein markers exhibited similar tendencies in terms of the order of intensity levels of protein bands, as shown in Figure 7B, regardless of the separation method. With respect to the level of exosomal protein markers, the methods can be arranged in the following order: PLL clustering > exoEasy > UC. These results confirmed that isolated ones are EVs contained surface (CD9 and CD81) and inner protein (TSG101 and ALIX) markers.

Total RNA was investigated in the different EV isolation methods. EV isolates were obtained from the same plasma, and RNA was extracted using the seraMir Exosome RNA purification kit. After RNA extraction, total RNA was analyzed on Agilent Eukaryote Total RNA Pico chips using a bioanalyzer kit chip and an Agilent bioanalyzer, as shown in Figure 7C. According to the RNA content, the methods can be arranged in the following order: PLL clustering > exoEasy > UC > ExoQuick. The results indicated that the PLL clustering method contained more enriched RNA than other EV isolation methods. In addition, the RT-qPCR experiment was conducted using hsa-let-7a-5p and hsa-miR-142-3p, known as miRNA markers of EVs [30]. In the analysis of Ct values, the present PLL clustering method showed similar values compared to UC, as shown in Figure 7D.

## 4. Discussion

We successfully isolated EVs from plasma using a cationic polymer (PLL) and a chaotropic salt (GuTc). This PLL clustering and chaotropic dissolution method is introduced for the first time in this paper, and it meets the core requirements of being fast, simple, and inexpensive. This method does not require any instruments, such as ultracentrifuges or even centrifuges; it only uses a syringe and a syringe filter at a very low cost. Furthermore, the entire process can be completed within 20 min.

Polymer-based EV-separation methods, such as ExoQuick, adopt precipitation and centrifugation by adding a polymer to the plasma. In the present PLL clustering method, a polymer was similarly added to the plasma, but precipitation and centrifugation were not necessary. Although conventional charge-interaction methods, such as exoEasy, adopt a stationary charged membrane, the present method adopts a freely moving charged polymer that can enhance the yield of EVs by capturing suspended nanoscale EVs in the liquid phase. Finally, our method only requires a single filtration step to exclude larger cells and debris and to filter the size-increased PLL clusters. Due tothe pore size (220 nm) of the filter used, one can easily operate dispensing and aspirating liquids. Therefore, combining charge interaction and chaotropic resuspension schemes is an innovative EV isolation method.

The essential feature of the present method is the introduction of a chaotic agent to dissolve PLL clusters and to resuspend EVs in the elution buffer. In this study, guanidinium thiocyanate (GuTc) was used as the most potent solubilizing chaotropic agent in the Hofmeister series [31]. It is known that chaotropic reagents denature proteins, thereby destroying the stability of the natural state of proteins and unfolding amino acid chains [32]. Since the clustering component, PLL, is a protein composed of amino acids (lysine), GuTc unfolds and dissolves the amino acid chains of PLL to allow resuspension of EVs in liquid. Surprisingly, the reaction of the chaotropic salt with PLL is very fast and active and, thus, flowing the GuTc solution over the PLL clusters is enough. These biochemical reactions drastically reduce the total time required to isolate EVs.

In a previous study, EVs were isolated by adding a mixture of protamine, a type of cationic polymer, and PEG, a low-concentration regent [33]; the study was successful in making clusters of EV and polymer, but the resuspension failed. Thus, the proposed solution was to minimize the polymer concentration to allow dissolution in PBS buffer. However, this solution led to a prolonged incubation time (>12 h) and a relatively poor yield. In this study, the difficulty in resuspending the polymer-induced clusters was easily solved by adopting chaotropic salts that removed exosomes from the cationic polymer.

In the filtration step of the PLL clustering method, washing, eluting, and EV recovery steps were conducted at a single membrane with a suitable pore diameter. In this study, we used a membrane with a pore size of 220 nm. When aspirating the plasma, large cells and cell debris greater than 220 nm in diameter were filtered. After mixing the filtered plasma with PLL and incubating it, PLL clustered particles larger than 220 nm remained on the filter. During eluting, resuspended EVs smaller than 220 nm passed through the membrane and were collected. By increasing the size of the targeted nanoscale EVs with PLL clustering, one can easily access and isolate them with a sub-micron mesh.

The selection of a proper washing reagent is highly important for removing proteins without damaging the isolated EVs. Thus, we examined the zeta potentials of various plasma proteins such as fibrinogen, globulin, and albumin (Figure S3). All of them yielded negative zeta potentials. It is worth noting that the zeta potentials of these proteins are smaller than those of the EVs except fibrinogen. If serum is the provided sample, the optimal concentration of GuTc washing buffer could only break the relatively weak bond between proteins and PLL, and purified EVs could be obtained. Thus, in the PLL clustering method, serum is preferred to plasma.

In the analysis of EV markers, we adopted two surface protein markers (CD9 and CD81) and two inner protein markers (ALIX and TSIG101). According to the recent guideline of Minimal Information for Studies of Extracellular Vesicles (MISEV), some proteins from non-EV structures would often co-exist in biofluids and, thus, negative protein makers, such as apolipoproteins A1/2 and B (APOA1/2, APOB), and albumin (ALB), were proposed for blood plasma and TammHorsfall protein (uromodulin/UMOD) for urine samples [34]. Unfortunately, the present study missed the negative markers, which should be carefully analyzed in further study.

EVs are important biomarkers; EV isolation is challenging owing to the inherent characteristics of EVs that include nanoscale size. Thus, EVs have required extremely high *g*-force UC, prolonged procedure times, and high total costs for preparation. However, using the characteristics of negative surface charge [25,30], EVs were easily isolated from plasma by adding a cationic polymer. PLL, a commonly used biocompatible cationic polymer, is a lysine homopolymer that has a positively-charged hydrophilic amino group of lysine. Due to this positive charge, PLL has been widely used as coating reagent to improve cell adhesion on the surfaces of culture dishes. Furthermore, PLL can be utilized to form DNA condensation using the electrostatic interaction between the negatively-charged backbone of DNA and the positively-charged domain of PLL. Likewise, cationic polymers and EVs can be clustered in a short time, as shown in Figure 1A, by simply adding PLL to the plasma sample.

Overall, the present PLL clustering method can be easily implemented as an integrated microfluidic chip that provides a sample-to-answer solution. Without adopting a sophisticated technique, a pipette tip equipped with a filter would be enough to apply the present method. If the provided samples are plasma and serum, their volumes could be less than a few milliliters, which can be sufficiently handled in a microfluidic chip or pipette tip. However, if the provided sample is an EV culture medium, which is tens of liters, the conventional kits may not be suitable. In fact, many industries extract exosomes from cell culture media for various applications, including targeted drug delivery. Surprisingly, the present method can be easily expanded to a massive volume operation. In our feasibility test, the cell culture medium included highly concentrated proteins, which were clustered with PLL without any cells. Again, the zeta potentials of these proteins are different from those of EVs, and thus, they can be washed out using an optimized GuTc washing buffer. Thus, the present PLL method, which is a rapid and simple way to isolate EVs, could accelerate basic research and clinical applications of drug delivery systems.

## 5. Conclusions

Despite the clinical significance of EVs, technical advances in EV isolation and downstream RNA and protein analysis have been remained a major challenge in the field of clinical oncology. UC has been set for a standard method of EV isolation in the research setting and clinical application of EVs has been extremely limited. Although commercial technologies including EV-precipitation or charge-interaction methods have contributed to somewhat operation convenience, it is still not satisfactory for use at clinical environments. Here, we introduce the novel EV isolation method, which is simple, quick, high-yield, and high purity isolation of EVs from blood plasma.

The present method has four key innovative features compared to the conventional methods for EV isolation including UC, polymer-precipitation, and charge-interaction methods. First, the present one is highly scalable to handle the sample volume from 10 μL to 50 L due to its unique characteristic of technology, whereas other methods have very limited sample volume up to several mL. However, pharmaceutical industry utilizing EVs as drug delivery system requires bulk sample volume processing in the range 20–100 L. The present method can freely handle large quantity of samples. Also, the present method can handle the sample volumes as small as 10 μL for point-of-care applications. Second, it provides 20 folds higher yields and 3.5 folds higher purity ratio compared to UC. Compared to commercial technologies, the present one has been found to be two folds to 20 folds higher performance in EV isolation. Third, it can be implemented as a size-selective isolation of EVs, which is getting important since EV sizes correlate with secreted cell type [35].

Furthermore, the present method provides many technical advantages and design flexibility over other EV isolation methods. First, it requires a single filter to exclude larger cells and debris and to filter the size-increased PLL clusters. Due to the pore size (220 nm) of the filter used, one can easily operate dispensing and aspirating liquids without any mechanical device. Additionally, the pore size of the membrane filter can be easily changed for a targeted EV size. Third, it does not require any bulky instruments such as ultracentrifuges or even centrifuges; it only uses a syringe and a syringe filter at a very low cost. Fourth, the entire process can be completed within 20 min, which would be the quickest operation among the existing technologies. Overall, the present method can be easily implemented as an integrated microfluidic system that provides a sample-to-answer solution. In our recent study, an automated microfluidic system (PIBEX) has been developed for extraction of cell-free DNA from plasma [36,37] In fact, the process of PIBEX for cfDNA extraction is quite similar to the present method, which can be implemented a chip.

In conclusion, we have successfully isolated and characterized EVs from blood plasma, which can provide molecular information in EVs. As a follow-up study, the present method will be used to isolate EVs to investigate genomic, transcriptomic, and proteomic information for potential applications in clinical oncology. This proposed method has the potential to accelerate EV-based biomarker discovery, molecular diagnosis and therapeutic monitoring, and drug delivery system, since it provides high quality output for EV isolation

## Supplementary Materials

The following are available online at https://www.mdpi.com/2077-0383/9/3/650/s1, **Figure S1.** EV cluster formation according to incubation time and temperature; The input PLL concentration was fixed at 0.5 mg/mL; **Figure S2**. Comparison of normal plasma and EV depleted plasma via pellet volume; **Figure S3**. Comparison of zeta potential results of representative plasma proteins: fibrinogen, globulin, and albumin.
