# Supplementary material for: Rapid and Efficient Isolation of Exosomes by Clustering and Scattering
Source: J Clin Med. 2020 Feb 28;9(3):650. doi: 10.3390/jcm9030650 (PMC7141250; doi:10.3390/jcm9030650)
Supplement: Supplementary file 1 [file jcm-09-00650-s001.pdf]

Supporting information for

# Rapid and Efficient Isolation of Exosomes by Clustering and Scattering

**Jinhyun Kim[1‡], Hoyoon Lee[1‡], KyongHwa Park[2,3], and Sehyun Shin[1, 3,*]**

[1] School of Mechanical engineering, Korea University, Seoul, Korea; mimoz742@korea.ac.kr (J.K.); leehoyoon@korea.ac.kr (H.L.)

[2] Department of Internal Medicine, Anam Hospital, Korea University, Seoul,02841, Korea; khpark@korea.ac.kr (K.H.P.)

[3] Engineering Research Center for Biofluid Biopsy, Seoul, 02841, Korea; lexerdshin@korea.ac.kr (S.S.)

‡ Jinhyun Kim and Hoyoon Lee contributed equally to the article as first authors

* Correspondence: lexerdshin@korea.ac.kr; Tel.: +82-10-4506-2825

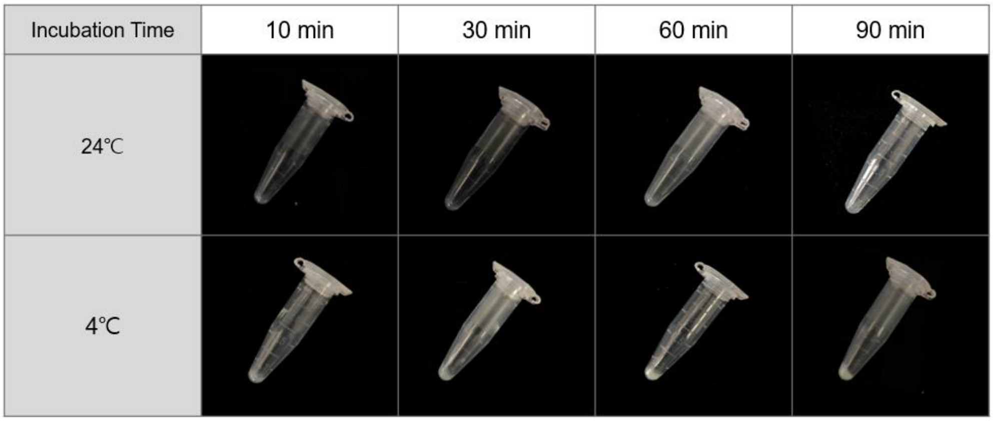


**Fig. S1** EV cluster formation according to incubation time and temperature; The input PLL concentration was fixed at 0.5 mg/mL.

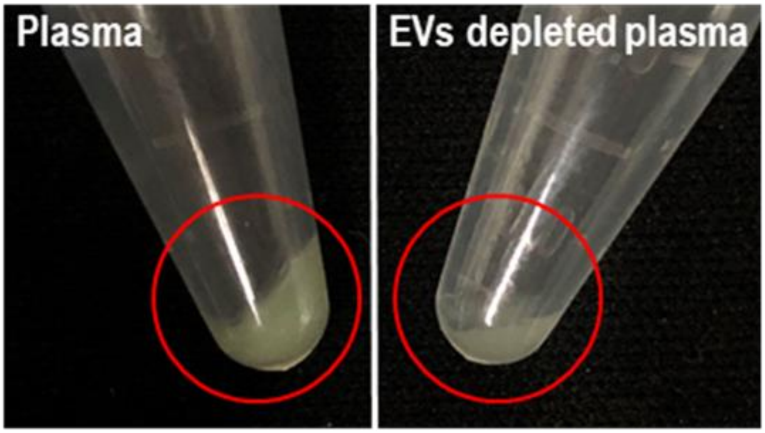


**Fig. S2** Comparison of normal plasma and EV depleted plasma via pellet volume

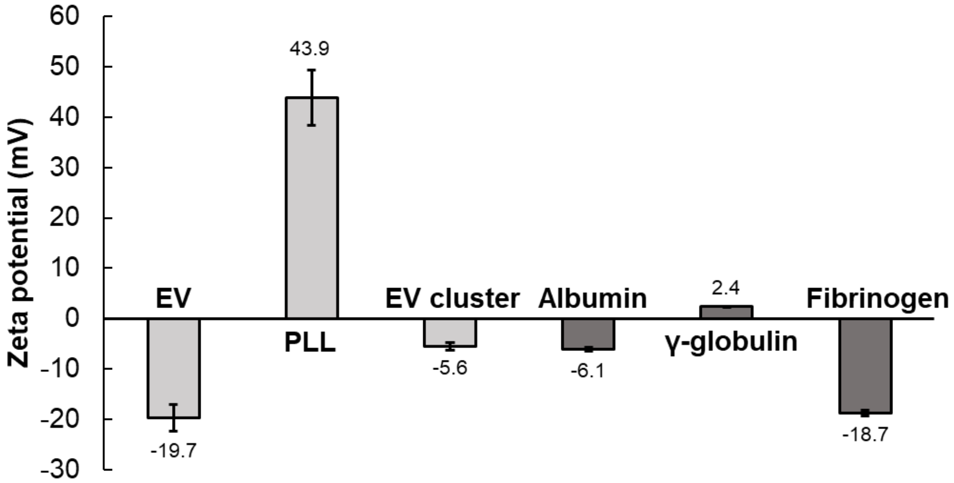


**Fig. S3** Comparison of zeta potential results of representative plasma proteins: fibrinogen, globulin, and albumin
